# Equitable Care for Children With a Tracheostomy: Addressing Challenges and Seeking Systemic Solutions

**DOI:** 10.1111/hex.14158

**Published:** 2024-07-26

**Authors:** Jules Sherman, Habib Zalzal, Kyle Bower

**Affiliations:** ^1^ Children's National Hospital, Innovation Ventures Washington DC USA; ^2^ Division of Otolaryngology Children's National Hospital Washington DC USA; ^3^ Department of Human Development and Family Sciences The University of Georgia Athens Georgia USA

**Keywords:** caregivers, healthcare, paediatrics, parents, policy, tracheostomy

## Abstract

**Background:**

Children with medical complexity (CMC) often face significant barriers to accessing care, obtaining appropriate insurance coverage for medical devices, technology, supplies, home nursing and social services. These challenges, when viewed through the lens of social determinants of health, highlight concerns about healthcare inequity. These inequities can impact CMC by limiting access to follow‐up appointments, leading to disproportionate use of emergency department services, restricting support services, reducing the quality of medical products and increasing the likelihood of adverse events. Addressing these concerns requires comprehensive policy changes at both state and federal levels. Achieving successful collaborations between states and federal agencies is particularly challenging and may take months or even years to accomplish.

**Objectives:**

Through an exploratory qualitative approach, this study facilitates a nuanced inquiry into the experiences and systemic challenges encountered by medical professionals and primary caregivers managing CMC who require a paediatric tracheostomy.

**Methods:**

Qualitative interviews were conducted with 17 health professionals and primary caregivers residing in the United States. A thematic analysis was used to analyse the transcribed interview data.

**Results:**

Using exploratory thematic analysis, we identified challenges and opportunities for improvement regarding (a) access to health insurance, (b) procurement of essential medical supplies, (c) logistical constraints and (d) identifying interim solutions.

**Conclusion:**

Building on our findings, we discuss how socioecological factors impact health and quality of life of CMC and families. Additionally, we address the growing gap in quality of care through a comprehensive approach that considers patient needs, regulatory frameworks and affordability.

**Patient or Public Contribution:**

Medical practitioners and healthcare professionals were actively involved in the development, production and implementation of the research project. These individuals were given the opportunity to review their statements and review the manuscript before publishing. While caregivers did not engage in member checking, each provided their consent before data collection.

## 1 Introduction

Paediatric tracheostomy is a life‐saving procedure for children with severe respiratory compromise or upper‐airway obstruction [1]. Approximately 4000 children in the United States undergo tracheostomy yearly, with a mean age of 3 years [2]. Most children with a tracheostomy represent a complex cohort of patients who require comprehensive and continuous medical attention. Throughout this paper, we will refer to this cohort as children with medical complexities (CMCs). To effectively manage their conditions and enhance their quality of life, children with a tracheostomy often require complex and coordinated medical care from multidisciplinary care teams [3, 4], as well as sustained reliance on specialized equipment, ranging from assistive devices to life‐sustaining technologies. However, navigating the complex landscape of procuring, funding and maintaining such equipment can be riddled with obstacles [5]. As a result, it is estimated that 60% of these children are readmitted to the hospital for a tracheostomy‐associated respiratory infection within the first 6 months of receiving a tracheostomy [6, 7].

Many families rely on social insurance programmes, such as Medicaid to supplement their existing healthcare plans. Medicaid is a healthcare programme in the United States that provides financial assistance to individuals and families in need. Jointly funded by federal and state governments, the programme is administered by individual states with oversight from the federal government. The programme provides a wide range of health services, including doctor visits, hospital stays, prescription medications, dental care, vision care and more. However, challenges faced by caregivers extend far beyond medical issues, encompassing social and environmental concerns [8, 9, 10]. Social determinants of health (SDoH), the socioecological factors that contribute to inequities that impact health and quality of life contextualize the barriers to healthcare (e.g., lack of health insurance, poor access to transportation and limited healthcare resources) [11]. Research examining SDoH has been influential in underscoring the socioecological factors contributing to the inequity experienced by patients and their families and outlining strategies for institutional change [12]. For instance, Sobotka et al. [13] found that frequent hospitalizations and emergency room visits result from financial limitations, bureaucratic hurdles, insurance complexities and the unique needs of each child. These factors collectively impact individual health, well‐being and quality of life. Although Medicaid is an important programme that provides access to healthcare for millions of Americans who might otherwise be unable to afford it, there are myriad restrictions and limitations, and the US healthcare system continues to frustrate patients, healthcare providers and families [5].

Although the contributing factors are still not fully understood [3, 4, 14], it is our aim to continue to raise awareness of the persistent challenges families face, particularly families of CMC in the context of SDoH. Through this paper, we aim to address the challenges within the healthcare system that create obstacles for caregivers, including issues with Medicaid and access to necessary equipment in various states, especially for families in rural or underserved areas. These concerns emphasize the urgency of policy reforms that address daily barriers to healthcare while also focussing on systematic change. Second, we highlight possible improvements to healthcare access and management for children with a tracheostomy and medical complexities. This involves enhancing the coordination of multidisciplinary care needed by these patient's families to manage their condition effectively.

## 2 Data and Methods

This explorative qualitative study emphasizes the experiences of primary caregivers, healthcare providers, administrators and legal counsel involved in caring for and supporting children with a tracheostomy. Participants were recruited using purposive and snowball sampling. Clinicians were recruited using purposive sampling through communication with leadership at Children's National Hospital (CNH) via email. They were included if they identified as clinicians, hospital administrators and legal counsel who specialized in providing support to paediatric patients with a tracheostomy and their families. Once we contacted key individuals, we asked if they had families they could refer. Eighty percent of the caregiver participants were contacted by physician referrals. Others were recruited through a Facebook support group for caregivers of CMC with tracheostomy. Caregivers were included if they were over the age of 18 years, a primary caregiver of a child with a tracheostomy and spoke fluent English.

Data collection began in March 2022 and concluded in November 2022, after obtaining IRB approval from CNH. The first author conducted individual interviews on a HIPAA–compliant Zoom platform with clinicians, hospital administrators and legal counsel. The semi‐structured interview guide outlined open‐ended questions (see Appendix I) that probed participants' perceptions of the US health system and their individual experiences of caring for children with a tracheostomy. Each participant was sent an e‐mail explaining the study along with a participant information sheet that outlined their participation in the study. Participants sent back their signed consent via e‐mail before a Zoom interview was scheduled.

Interviews were transcribed using the Zoom transcription tool. Participants were given a pseudonym name to maintain confidentiality while also indicating their professional title (e.g., Dr. Adani, Rhea, RN). The de‐identified transcripts were then uploaded to ATLAS.ti 22 for Mac, which is qualitative data analysis software (QDAS) used to assist with data management and analysis [15, 16]. We used the coding tool and commenting tool in ATLAS.ti to identify patterns in the data. Due to the exploratory nature of this study, the analysis began with open coding, which captured the descriptive characteristics of quotations (e.g., data). Throughout our analysis, the team used ATLAS.ti memos to reflect on the data and audit the progression of our discussions, which in turn informed our interpretations of the data [15]. Codes were then grouped into larger themes (e.g., axial coding) using code groups in ATLAS.ti [16]. To further ensure the reliability of the interpretive analytical process, data were returned to participants so they could check for accuracy and confirm statements resonated with their experiences (i.e., member checking) [16]. Of those who responded to the request, only one participant requested minor changes, which were integrated into the analysis and subsequent results. The findings illuminate critical challenges facing healthcare providers who care for children with tracheostomies.

## 3 Results

We recruited 17 participants. Six participants self‐identified as the primary caregiver to their child with a tracheostomy. Their ages ranged from 27 to 55 (*M* = 36). One female participant identified as a Latina grandmother, and she was also the only participant who did not identify as White. Only one mother was divorced at the time of the interview. The other 11 participants were paediatric doctors/specialists (*n* = 3), nurses (*n* = 4), a reimbursement expert (*n* = 1) and health service providers (e.g., integrated care managers and a social worker) (*n* = 3). These individuals were from various racial backgrounds. Notably, income data was not collected for participants as it was not important to the scope of the project. Clinicians described their practice as large (i.e., serving 1000+ families per year). Participants serve diverse communities, meaning they see and work with people who are impacted differently by the SDoH. The 17 participants live (and practice) in six US states. Table 1 summarizes the heterogeneity of the recruited sample.

**Table 1:** Participant characteristics.

Pseudonym	State	Role
Dr. Adani	Washington, DC	Medical specialist and vice president of a children's medical specialty centre
Dr. Farid	Washington DC	Medical specialist and faculty of otolaryngology
Dr. Jagger	Washington, DC	Paediatrician; hospital administrator
Rhea, RN	Washington, DC	Clinical nurse manager
Kelly, RN	Washington, DC	Nurse
Margaret, RT	California	Business owner; patient advocate and respiratory therapist (RT)
Nadia, RN	Washington, DC	Integrated care director
Heidi, RN	Washington, DC	Surgical airway and tracheostomy specialist
Leiya, RN	Washington, DC	Ambulatory case manager for children with medical complexity
Marlowe, MS	Washington, DC	VP of revenue cycle and care management
Brian, JD	Washington, DC	President of a medical reimbursement strategy firm
Brooke	Nebraska	Mother of a 3‐year‐old tracheostomized girl
Lania	Illinois	Grandmother of a 2‐year‐old tracheostomized girl
Anne	Florida	Mother of a 16‐month‐old tracheostomized girl
Justine	California	Mother of a 2‐year‐old tracheostomized son
Carla	Virginia	Mother of a 1.5‐year‐old tracheotomized son
Doug	Virginia	Father of a 4‐year‐old tracheotomized son

The following results demonstrate how gaps in health insurance coverage, restrictions on necessary medical equipment supplies and logistical challenges contribute to frustrations experienced by primary caregivers of CMC and professionals in the healthcare industry. Within each section, we triangulate the meaning of the theme through participants' differing perspectives (e.g., the caregiver, the provider and the medical professional), shedding light on the pervasiveness of particular issues. Furthermore, although we present Themes 1–3 as separate challenges, the fourth presents how participants' organizations are addressing the gaps in health policy and coverage using comprehensive and solution‐focussed strategies.

### 3.1 Navigating Complexities of Health Insurance

In the United States, CMCs receive coverage through state‐based programmes, including enrollment in a Medicaid managed care (MMC) or a fee‐for‐service (FFS) plan. However, even when paediatric tracheostomy patients have health insurance, variations in coverage create barriers to equitable care. Given the bureaucracy within and among states, caregivers and providers struggle to identify ways to support children that effectively address their immediate need for assistance. Nadia, RN and an integrated care director, stresses the glaring social issue of inequitable health care experienced by millions of families across the United States:

I work with families who have social determinants concerns. These families have financial issues where the mom is having to make the choice between, ‘Do I keep the phone on, do I buy gas to get to the hospital, or something else for the child?’

She goes on to explain:

I would say the thing that I find the most challenging is knowing that a family has a need but understanding that the way that the system is set up, I might not be able to meet that need adequately. The resources through Medicaid may not be there, or there are major restrictions. There are always socio‐economic issues with our population, so I try to make things work as well as I can, but sometimes, it is just the way that things are set up in the Medicaid system, and I am powerless.

Several participants shared their irritations with the US health system and the multiple layers of policy and procedure caregivers must endure. Margaret, RT, captures similar instances saying:

Parents should not have to beg, steal, or borrow for the supplies they need to take care of their kids. The parents I know are getting four trach‐ties per month, so, they must wash them. The ties are not meant to be washed. They are disposable. They fall apart. It's not safe…. DME companies will not go the extra mile, so they will say things are ‘discontinued,’ but they just need to make a few extra calls to obtain a product. It's frustrating to parents because they cannot get what they need, and there are no substitutions. They cannot get what they need to keep their children breathing correctly! To be told ‘it's not available,’ well, that is not an option!

Caregivers also noted that these gaps in coverage led to more significant health issues for their children, including difficulties in scheduling routine medical appointments and fears about inaccessible emergency care. Dr. Farid explains the complexity from the perspective of a medical provider:

If [DMEs] do not give the patient what they need and something happens to the patient, the doctor is technically liable, even though it was not our product recommendation. I don't think [distributing inadequate products] is fair to the patients, or fair to the physicians. Honestly, I don't think it's fair to the entire healthcare system for a payer or DME to look at it from a cost management perspective when we are talking about providing quality life‐sustaining equipment at home.

Several participants emphasized the importance of establishing consistent and comprehensive health insurance coverage for paediatric patients with a tracheostomy to address existing gaps in coverage.

### 3.2 Procurement of Essential Medical Supplies

Participants were forthcoming in describing the nuances of health coverage and medical service procurement as coverage determines the availability of specific services and equipment. Brian, JD and the president of a medical reimbursement strategy firm, outlines the administrative challenges that have compounded over time:

Most Medicaid payment levels (for medical products) were set 10–20 years ago. Since then, there have been many new products, innovative technologies, that improve net health outcomes, but are usually more expensive. There are, however, limited mechanisms and no incentives for state Medicaid programs to pay more for more efficacious equipment. This is frequently the case for private payers too.

He goes on to explain:

A doctor sends a prescription for a new device to be used in the home to the Durable Medical Equipment (DME) supplier. That DME supplier submits a claim to the patient's health insurer and bills with a code for devices that were created and priced 10–20 years ago. Many coding and pricing authorities do not recognize innovative new devices. Payment levels are old and inappropriate for innovative devices. The DME supplier knows they will receive only the ‘old’ payment levels and have no incentive to fill the prescription with the innovative device. DME suppliers need high volume, not innovation, to sustain their business model. Outdated reimbursement policies, particularly for high tech home care, create barriers to high quality care.

Medical professionals also provide important background regarding how insurance reimbursement works in the medical device world. They call attention to the problematic disincentivized medical system and outdated reimbursement structure.

Dr. Adani says, ‘Systematic problems call for systematic change’, and goes on to explain:

We can advocate all we want, but insurers do what they want. That's even for tracheostomies. We know that a trach (tracheostomy tube), should be changed every week. Some payers (insurers) only supply one per month. What are families supposed to do? It is ridiculous. They clean their own trachs, they sanitize their own trachs. It is the most ridiculous thing. It is crazy, I mean, what can we do? But that is not for you or me to decide. That problem is at a policy level.

The inflexibility of policies further complicates scenarios that are already life threatening. For instance, Lania, a grandmother of a 2‐year‐old tracheostomized girl in Illinois, shares an example of how little flexibility exists, even when circumstances are beyond their control:

Last year in July, we lost power for almost two weeks when we had a storm that knocked down the electrical pole for our house. Because she is also on a G‐tube, her formula must be kept cold in the refrigerator, and of course her oxygen concentrator needed to be running during the night, so we ended up having to purchase a generator, since we needed to keep her medicines and formula cold and run the oxygen. I am still paying off that (generator) bill because Medicaid has not covered the cost.

The testimony presented here emphasizes a critical gap between the advent of innovative medical devices and the outdated reimbursement models that fail to support them. Systematic reform is essential to align insurance coverage with medical advancements, ensuring that children with a tracheostomy receive the care they need without undue financial strain on their families.

### 3.3 Logistical and Transportation Constraints

Within this theme, parents and healthcare professionals describe the unique challenges of transporting a CMC for essential medical care. Although parents and caregivers understand the necessity of follow‐up care to experience the best possible health outcomes, they portray limited access to safe and reliable transportation. Rhea, RN, is aware that the issue of attrition is not as simple as no‐shows or missing appointments:

Some of our patients have been lost to follow‐up appointments for some years, and they only think about it if there are health issues. However, I think many families have transportation challenges because of logistics, or where they live.

Two caregivers, Anne and Carla, provide further insight as neither has reliable access to medical transport services. Anne explained:

Driving to appointments by myself is scary. I cannot do it by myself, which is why I need a nurse to sit in the backseat.

She went on to say that her husband is not comfortable sitting in the backseat, solely responsible for their daughter because he is too nervous something could go wrong. Carla concedes:

I am not comfortable with doing more than a 15‐minute drive (with her son) without having the nurse with me. Most of our appointments are a state away, so we book multiple appointments in one day and end up spending 7–8 hours there. It is not easy to plan those full days away from home.

Dr. Adani also comments on the impact logistical constraints have on treatment. He reflects on his own medical practice, adding:

At the root of the problem is the family's inability to show up for their six‐month appointments, and this is usually an access issue. If they showed up for their regular appointments, then maybe we could troubleshoot issues earlier (to avoid emergency scenarios) but people are not showing up to appointments. That is a big piece of the puzzle.

Furthermore, while planned medical visits pose several challenges, such as scheduling constraints and managing the child's needs during the trip to a medical facility, parents also discussed emergency scenarios. Doug, the father of a 4‐year‐old in Virginia, explains what happens when his daughter needs immediate medical attention:

We are an hour and a half away from our hospital, so we end up having to be taken by ambulance or by helicopter if there is an emergency simply because it takes too long to get him (our son) there in time on the freeway. There have been multiple helicopter flights.

Knowing they may be unable to access life‐saving medical care for their child during the next emergency increases anxiety among parents with children who have a tracheostomy. At the same time, medical professionals grow frustrated with the knowledge that many emergency scenarios are avoidable with routine care and attending follow‐up appointments. Both groups feel the constraints of a healthcare system that does not adequately address the origin of the issues, leading to poorer outcomes for children and families.

### 3.4 Identifying Interim Solutions

The solutions shared by participants are in stark contrast. On the one side, participants shared their workarounds that are not ideal but essential for their child's survival. The other side is endeavouring in grassroots efforts that carve a pathway for lasting change.

When systematic change is unattainable, families and practitioners seek short‐term resolutions to help defer the crises and minimize chaos. In the next three quotes, caregivers provide insight into the difficult decisions they must make, and while these are not medically advisable, their priority is to keep their child alive. Anne, a mother of a tracheostomized child born with congenital central hyperventilation syndrome living in Florida, says:

Last month, when we called in our order, the service person at the DME said she could only send me two trachs (tracheostomy tubes). I told her that we normally get four per month, and to please check our previous orders. I knew they were going to try to short me this month. It all depends on who you talk to, because sometimes they (the DME) will just let it go. But I worry about the lack of life‐saving supplies being covered by insurance and I'm tired of arguing about the requirements set by the doctors.

Brooke, a mother of a 3‐year‐old tracheostomized daughter living in Nebraska, says:

We have trach ties we found that are much better [‘Marpac Comfort Collar’]. With these new ties there is no need for shoving the Absorbx around her stoma or the 2 × 2 gauze. They [‘Marpac Comfort Collar’] have built‐in absorbing pads. Sadly, our DME does not offer them, so they are not paid for by insurance. I try to get them from our hospital, and from Facebook groups that trade or give away supplies.

The other part of this issue is the lengths to which parents and caregivers will go to secure supplies and resources when they are not readily available. Lania, a grandmother of a 2‐year‐old tracheostomized girl in Illinois, says:

We resort to exchanging supplies through black market Facebook groups and reusing trachs (tracheostomy tubes), which we boil in water, air dry, and store in vacuum‐sealed bags. A distilled water shortage is another challenge we face. While we try our best to sanitize and reuse trachs, it concerns me that there are risks involved, and I wish we could get new ones every week to avoid potential complications.

In the absence of systematic health reform, some clinicians are identifying innovative solutions to meet urgent needs. These participants shared how they built their own solutions through internal hospital or regional grants. One example is Heidi's (a surgical airway and tracheostomy specialist) parent educational booklet called ‘Trach Me Home’, designed to guide parents caring for a child with a tracheostomy. Dr. Jagger discussed the CNH Parent Navigator Programme, which is designed to be comprehensive and assists families whose children require frequent doctor visits, home nursing care, multiple hospital stays, consultations with several specialty doctors, specialized education (e.g., individualized education plan or individualized family service plan), various therapy services (such as physical, occupational or speech therapy) or the use of medical equipment like wheelchairs, ventilators, feeding tubes or computer‐aided technology.

In response to the complexity of integrated health care for CMC, participants also highlighted community‐based programmes that address transportation constraints commonly experienced by families with CMC. These are local programmes that offer subsidized transportation, collaborative ride‐sharing services and mobile clinics. Furthermore, a significant issue for families of CMC is learning how to care for their child when they are discharged from the hospital. To address the concerns, Nadia, RN, described a programme at HSC (Hospital for Sick Children) Paediatric Center that was developed to ease the transition from the hospital to the patient's home:

In our facility, we have an apartment for parents to stay in while they learn how to care for their child at home. This is really a benefit to patients and families because it makes it more real. They have the resources available, like calling an [on‐call] nurse if there is an issue. In some states, they don't have any kind of intermediate care facility. They [patients and families] must go to another state if the child and family needs this.

These efforts highlight the ongoing challenges and the innovative ways in which organizations are addressing the gaps in health policy and coverage. As these programmes continue to make a significant impact, their success stories support the adoption of integrated health models in children's hospitals nationwide.

## 4 Discussion

Health insurance and Medicaid, durable medical equipment (DME) companies and logistical concerns, present multifaceted challenges that encompass both systemic and individual constraints [5]. Throughout our findings, participants indicated how socioecological factors impact health and the quality of life of their patients and families. Furthermore, their statements were not dissimilar from existing research that also underscores a push for healthcare reform that prioritizes the health and well‐being of medically complex children and their families. To mitigate the growing gap in quality of care, scholars and medical professionals have called for standardization of practice that accounts for the inequity that exists in the United States healthcare system [11, 14, 17]. We can close significant gaps by lobbying for broader coverage and advocating for innovative funding models such as bundled payments, which involve a single, comprehensive payment for all services related to a specific treatment or condition over a defined period. This model contrasts with traditional FFS payments, which bill separately for each service provided. Addressing these issues requires a broad approach that considers patient needs, regulatory frameworks, market dynamics and affordability.

Problems with access to medical supplies have significant policy implications for healthcare systems, governments, regulatory bodies and healthcare providers. For example, one participant, who is a medical reimbursement professional, suggested updating insurance codes to correspond with products that reflect current technological standards, reducing the financial burden on families. The participant explained that this could be achieved by establishing a national standard for medical device coverage for children, which would streamline the approval process and eliminate inconsistencies among different insurers. His comments are consistent with existing policies outlined by the Institute of Medicine, which recommends reviewing research prioritizations every 3–5 years to inform improvements in healthcare delivery and technologies [14]. By evaluating systematic factors that control access to these critical supplies and services, we, as a society, can better understand the social barriers that prevent CMC from receiving the care they need to manage their conditions effectively [18, 19], and thus, reducing the need for hospitalizations and improving long‐term outcomes. Integrated care programmes are more than just a service; they represent a movement toward a compassionate, family‐centred approach that addresses the extensive needs of children with a tracheostomy and their families.

Safe and reliable transportation to and from appointments is an essential component of the integrated care model, particularly for families without access to a vehicle or living in areas with poor public transportation. For instance, a respiratory therapist emphasized parents' fears and difficulties when travelling alone, while others emphasized logistical challenges that lead to patients missing follow‐up appointments. To mitigate logistical challenges, integrated care partners (e.g., clinicians, specialists and social workers) are exploring the use of telemedicine and its role in preventing adverse tracheostomy and ventilator‐related events [20, 21]. Studies suggest that telehealth is increasingly instrumental in providing access to healthcare for patients who may not be able to physically visit a healthcare facility [22, 23, 24, 25]. This is particularly important for follow‐up appointments, which often require less in‐person interaction than initial consultations. Remote healthcare technology can take many forms, including telemedicine, virtual consultations and remote monitoring devices. By utilizing advanced remote healthcare technology, healthcare providers can ensure that follow‐up appointments are more accessible to patients, especially those who live in rural or remote areas, have mobility issues or have difficulty scheduling appointments during regular business hours [26, 27]. Although telemedicine has led to improved surveillance of paediatric patients who may live far from their provider's office [28], its role as it relates to paediatric tracheostomy patients requires more research, as their care requires a multidisciplinary approach [29].

Given the findings and existing literature, there is an overarching aim to foster a US healthcare system that is responsive to home‐based care needs and the integral medical devices that safeguard children's health. The discordance between state Medicaid regulations and the continuity of care for CMC necessitates harmonization. Advocacy efforts should push for state regulations that mirror federal guidelines, thereby eradicating inconsistencies that jeopardize care standards. Furthermore, federal legislation can serve as a model for states, guiding them towards uniformity and ensuring no child's care is compromised by their zip code [30]. Addressing health insurance companies' entrenched tactics and policies requires a blend of advocacy for policy reform and collaborative engagement. Outdated provisions need revisiting due to evolving paediatric care standards and new medical technology innovations. One concrete way we can forge a better future is to take advantage of a new US Food and Drug Administration (FDA) initiative, ‘Health Care at Home’, which focusses on advancing health equity [30]. FDA's initiative supports medical device testing that enhances patient care, empowers caregivers, reduces the need for hospital readmission and ultimately contributes to a more equitable healthcare system. Further, proactive dialogue with insurers, supported by robust data that elucidate the long‐term benefits and cost‐effectiveness of ‘hospital at home’, will be vital in this endeavour.

## 5 Conclusion

The issues presented throughout this paper are not isolated from one another. Rather, they emphasize the complex network of barriers that contribute to the inequity in healthcare for CMC who have a tracheostomy. This study was exploratory; therefore, the transferability of findings is limited by the characteristics of the people in our sample. As discussed, integrated healthcare is an interprofessional approach to addressing the health needs of patients and supports a more holistic and tailored approach to patient care. There is a growing body of research showing the benefits of this approach, including improved physical, mental and emotional well‐being [31]. Furthermore, comprehensive policy changes to healthcare and the medical industry are necessary at both the state and federal levels, but collaborations among states and federal agencies take months, if not years, to establish. Policy moves too slowly for families reusing supplies and equipment, negotiating coverage and deciding whether to pay household or medical bills. Although this research focussed specifically on paediatric patients facing challenges related to a tracheostomy, the issues identified are undeniably relevant to a wider range of complex paediatric medical conditions.

## Author Contributions

**Jules Sherman:** conceptualization, investigation, writing–original draft, writing–review and editing, formal analysis, supervision, resources, software, project administration. **Habib Zalzal:** validation, writing–review and editing. **Kyle Bower:** methodology, formal analysis, writing–original draft, software, project administration.

## Ethics Statement

This study was approved by the Children's National Hospital Office for the Protection of Human Subjects (STUDY00000094).

## Conflicts of Interest

The authors declare no conflicts of interest.

## Data Availability

The data that support the findings of this study are available on request from the corresponding author. The data are not publicly available due to privacy or ethical restrictions.

## Appendix I

1. Where do you work or provide care?
2. How many years of experience do you have?
3. Describe how you support tracheostomy patients (integrative care programmes).
4. How many tracheostomy patients do you work with every month?
5. Describe everyday use and issues with tubes and care (e.g., install, maintenance, monitoring, training).
6. What are the main problems you manage in the hospital for patients with a tracheostomy?
7. Describe the training you offer for parents before discharge.
8. Can you tell me a story that highlights a common emergency?
9. What brand of tracheostomy supplies do you work with?
10. What are the key issues with the equipment you are working with?
11. How are you attempting to solve these issues?
12. Describe DME companies and how you interface with them.
13. What programmes are in place at the local, regional and national levels for CMCs?
14. What are the challenges families face with caring for their CMC at home?
15. What does the insurance landscape look like for CMC, and what problems do parents experience with DME companies to obtain what they were prescribed? (for medical regulatory legal expert)
